# Comparison of droplet digital PCR and direct Sanger sequencing for the detection of the *BRAF* ^V600E^ mutation in papillary thyroid carcinoma

**DOI:** 10.1002/jcla.22902

**Published:** 2019-04-25

**Authors:** Zhuo Wang, Kejing Sun, Changwen Jing, Haixia Cao, Rong Ma, Jianzhong Wu

**Affiliations:** ^1^ Clinical Cancer Research Center, Jiangsu Cancer Hospital & Jiangsu Institute of Cancer Research & The Affiliated Cancer Hospital of Nanjing Medical University Nanjing China; ^2^ Genesmile Company Nanjing China

**Keywords:** *BRAF*^V600E^ mutation, ddPCR, papillary thyroid carcinoma, Sanger sequencing

## Abstract

**Background:**

The *BRAF* ^V600E^ mutation status is a useful diagnostic and prognostic marker for papillary thyroid carcinoma (PTC). Although it is a commonly used method, Sanger sequencing has several limitations in detecting the *BRAF* ^V600E^ mutation. The aim of this study was to evaluate the efficiency of droplet digital PCR (ddPCR) as an alternative method for the detection of the *BRAF* ^V600E^ mutation in PTC patients.

**Methods:**

Samples from a total of 120 patients with PTC and 30 patients with benign nodular thyroid disease who underwent thyroid surgery were collected. The *BRAF* ^V600E^ mutation status of the PTC patients was tested by Sanger sequencing and ddPCR.

**Results:**

The *BRAF* ^V600E^ mutation was detected in 67 samples (44.67%) by Sanger sequencing and 92 samples (61.33%) by ddPCR. The detection of the mutation by the two methods was inconsistent in twenty‐five samples (16.67%). The sensitivity and specificity of the ddPCR method were 100% and 69.88%, respectively, and the positive predictive and negative predictive values were 72.83% and 100%, respectively. The concordance rate between the two methods in detecting the *BRAF* ^V600E^ mutation was 83.33%. Neither Sanger sequencing nor ddPCR detected *BRAF* ^V600E^ in 30 patients with benign nodular thyroid disease. The 92 samples with the *BRAF* ^V600E^ mutation were detected by ddPCR at a fractional abundance from 0.28% to 45.40% as follows: ≥10% (59 samples, 64.13%), 5%‐10% (8 samples, 8.70%), and ≤5% (25 samples, 27.17%). The *BRAF* ^V600E^ mutation was detected in all 59 samples at a fractional abundance ≥10% and in four samples at a fractional abundance from 5% to 10%, and no *BRAF* ^V600E^ mutation was detected at a fractional abundance ≤5% by Sanger sequencing.

**Conclusions:**

ddPCR was a reliable, highly sensitive alternative method for the detection of the *BRAF* ^V600E^ mutation in PTC patients.

## 1 INTRODUCTION

Thyroid cancer (TC) is the most common type of endocrine malignancy, and papillary thyroid carcinoma (PTC) accounts for the vast majority (90%) of thyroid malignancies.^1^, ^2^ *BRAF* ^V600E^ has a T1799A point mutation in exon 15, resulting in the substitution of valine for glutamic acid at amino acid 600 (V600E), and it is the most common driver mutation of PTC. In addition, the *BRAF* ^V600E^ mutation is associated with aggressive clinical features and a higher risk of recurrence of PTC^3^ and may helpful in detecting malignancy in some thyroid nodules.^4^, ^5^

Many techniques can be used for the detection of the *BRAF* ^V600E^ mutation,^6^, ^7^, ^8^ and the gold standard method for the molecular diagnosis of the *BRAF* ^V600E^ mutation is direct Sanger sequencing.^2^ However, Sanger sequencing identified only 7%‐20% of mutated alleles in a background of wild‐type alleles.^8^, ^9^

Droplet digital PCR (ddPCR) is a recently introduced technology that may facilitate the detection of rare point mutations in a background of wild‐type alleles. The ddPCR technique is based on the partitioning of the sample into thousands of microreactions of a defined volume. After PCR, each droplet either does or does not contain the nucleic acid of interest, allowing estimation of the number of molecules in the reaction under the assumption of a Poisson distribution. The results are expressed as target copies per microliter of the reaction volume.^10^

The limit of detection of ddPCR is 0.0005%.^11^ Therefore, ddPCR analysis sensitively detects and quantifies low‐abundance gene mutations like the *BRAF* ^V600E^ mutation.

In the present study, the *BRAF* ^V600E^ mutation was detected by ddPCR and Sanger sequencing. ddPCR was a better method than Sanger sequencing in detecting the *BRAF* ^V600E^ mutation in PTC samples.

## 2 MATERIALS AND METHODS

### 2.1 Patients

A total of 150 patients who underwent thyroid surgery between November 2016 and February 2019 in the Jiangsu Cancer Hospital were included in this study. All specimens were obtained after thyroid surgery, fixed in formalin, and embedded in paraffin. These samples were from 120 patients with PTC and 30 patients with benign nodular thyroid disease.

Tumor staging was performed according to the seventh edition of the tumor‐node‐metastasis (TNM) classification by the American Joint Committee on Cancer (8th edition). All patients who participated in the study gave their informed consent.

### 2.2 DNA extraction

Hematoxylin‐eosin slides prepared from specimens were examined by two experienced pathologists to estimate the areas enriched in tumor cell populations. Tissue was scraped from this preselected area and transferred to an Eppendorf tube for DNA extraction using the QIAamp FFPE Tissue Kit (Qiagen).

### 2.3 Direct Sanger sequencing to detect the *BRAF* ^V600E^ mutation

Using the forward primer 5′‐CTCTTCATAATGCTTGCTCTGATAGG‐3′ and the reverse primer 5′‐AGTTGAGACCTTCAATGACTTTCTAGT‐3′, exon 15 of the *BRAF* gene, which potentially contained the T1799A transversion mutation (encoding *BRAF* ^V600E^), was amplified by PCR. Amplification was performed under the following conditions: 1 cycle at 95°C for 5 minutes, 35 cycles of denaturation at 95°C for 30 seconds, annealing at 55°C for 30 seconds, and extension at 72°C for 40 seconds; followed by a final extension at 72°C for 5 minutes using Premix Taq™ Hot Start Version (Takara). The purified PCR products were sequenced using the forward primer above and a BigDye Terminator v 3.1 kit (Thermo Fisher). Capillary separation and data collection were performed on an ABI 3500 Genetic Analyzer.

### 2.4 ddPCR analysis to detect the *BRAF* ^V600E^ mutation

The ddPCR mixtures, 20 μL in volume, contained 20 ng DNA, 1× ddPCR supermix for the probe (no dUTP), 0.5 μmol/L of each primer (forward primer: 5′‐CATGAAGACCTCACAGTAAAAATAGGTGAT‐3′ and reverse primer: 5′‐ TGGGACCCACTCCATCGA‐3′), and 0.25 μmol/L of each probe (wild type: 5′‐VIC‐CGAGATTTCACTGTAGCT‐MGB‐3′, mutation: 5′‐FAM‐CGAGATTTCTCTGTAGCT‐MGB‐3′). Droplets were generated and analyzed using the QX200 system (Bio‐Rad). Amplification was performed as follows: 95°C for 10 minutes (1 cycle), 94°C for 30 seconds and 55°C for 1 minutes (40 cycles), and 98°C for 10 minutes (1 cycle) with a ramp rate of 2°C/s, and the reaction was then held at 4°C with a ramp rate of 1°C/s. The absolute quantification of mutant alleles and wild‐type alleles by ddPCR was estimated by modeling as a Poisson distribution using QuantaSoft v1.6.6 analysis software (Bio‐Rad). The threshold was defined as that described in the “Droplet Digital Application Guide.” Samples with a droplet number of more than 3 in the positive area were considered positive by the QuantaSoft analysis software.

## 3 RESULTS

### 3.1 Clinical characteristics

The clinical characteristics of the patients are shown in Table 1. Of the included patients, 62 (41.33%) were male and 88 (58.67%) were female, and the average age was 57 years (ranged from 17 to 72).

**Table 1:** The clinical characteristics of patients

Characteristics	Patients with PTC	Patients with benign nodular thyroid disease	Total
Age			
≥55	37 (30.83%)	12 (40.00%)	49 (32.67%)
<55	83 (69.17%)	18 (60.00%)	101 (67.33%)
Gender			
Male	51 (42.50%)	11 (36.67%)	62 (41.33%)
Female	69 (57.50%)	19 (63.33%)	88 (58.67%)
pT stage			
T1	59 (49.17%)		
T2	11 (9.17%)		
T3	38 (31.67%)		
T4	12 (10.00%)		
pN stage			
N0	59 (49.17%)		
N1	61 (50.83%)		
AJCC cancer stage, 8th edition			
I	89 (74.17%)		
II	27 (22.50%)		
III	4 (3.33%)		
IV	0		

### 3.2 Comparison of Sanger sequencing and ddPCR detection of the *BRAF* ^V600E^ mutation

**Figure 1:**
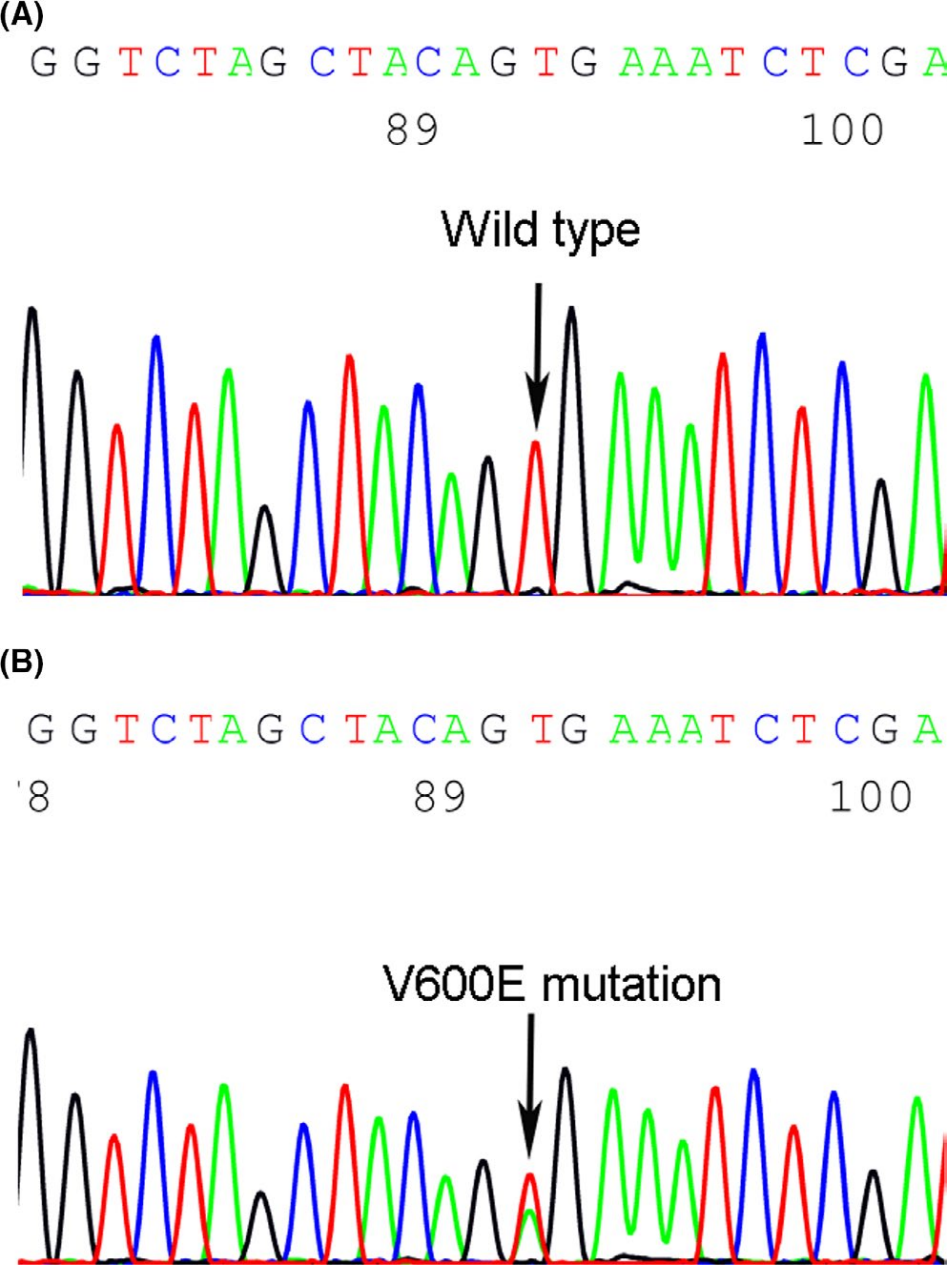
A, The representative wild type of *BRAF* ^V600E^ detection in papillary thyroid carcinoma (PTC) by Sanger sequencing. B, The representative *BRAF* ^V600E^ mutation in PTC by Sanger sequencing

**Figure 2:**
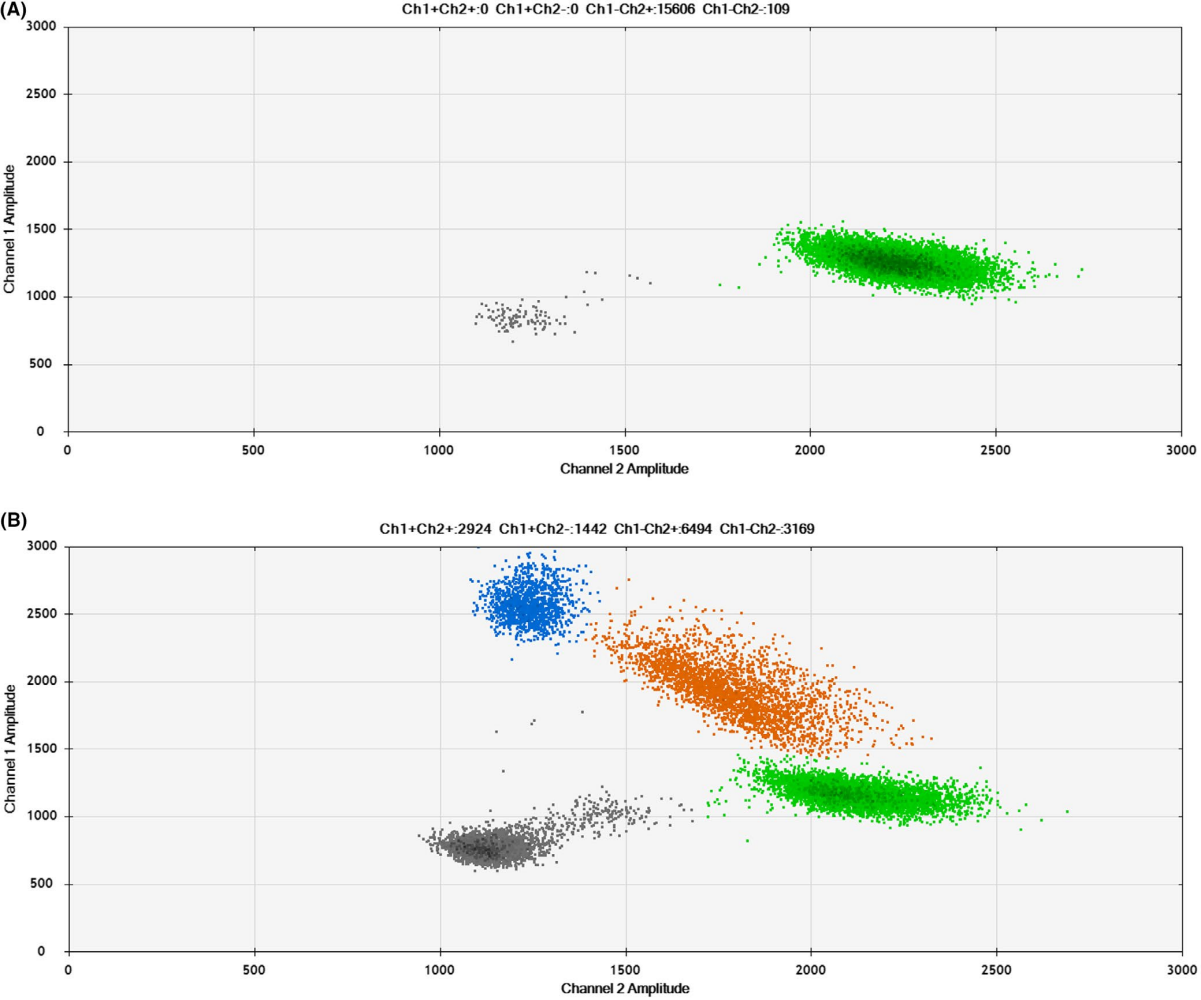
A, The representative wild type of *BRAF* ^V600E^ detection in papillary thyroid carcinoma (PTC) by ddPCR. B, The representative *BRAF* ^V600E^ mutation in PTC by ddPCR. Blue and orange dots represent the presence of mutant DNA

Table 2 shows the results of Sanger sequencing and ddPCR for the detection of the *BRAF* ^V600E^ mutation in 150 patients. The *BRAF* ^V600E^ mutation was detected in 67 samples (44.67%) by Sanger sequencing and 92 samples (61.33%) by ddPCR. Of these samples, the detection of the mutation in 25 samples (16.67%) by the two methods was inconsistent. All 25 samples were identified as having the *BRAF* ^V600E^ mutation by ddPCR and wild type by Sanger sequencing. No false negatives were detected using the ddPCR test. Figure 1 shows the representative figure of *BRAF* ^V600E^ detection in PTC samples by Sanger sequencing, and Figure 2 shows the representative figure of *BRAF* ^V600E^ detection in PTC samples by ddPCR.

**Table 2:** Comparison of the results of Sanger sequencing and ddPCR for *BRAF* ^V600E^ in 150 patients

ddPCR	Sanger sequencing		Total
	*BRAF* ^V600E^	Wild type	
*BRAF* ^V600E^	67 (100%)	25 (30.12%)	92(61.33%)
Wild type	0	58 (69.88%)	58(38.67%)
Total	67 (44.67%)	83 (55.33%)	150(100%)

The sensitivity and specificity of the ddPCR method and Sanger sequencing were 100% and 69.88%, respectively, and the positive predictive and negative predictive values were 72.83% and 100%, respectively. The concordance rate between the detection of the *BRAF* ^V600E^ mutation by ddPCR and Sanger sequencing was 83.33%.

The *BRAF* ^V600E^ mutation was found in 55.83% of PTC patients by Sanger sequencing and 76.67% by ddPCR. Neither Sanger sequencing nor ddPCR analysis detected the *BRAF* ^V600E^ mutation in 30 patients with benign nodular thyroid disease (Table 3).

**Table 3:** The results of *BRAF* ^V600E^ in patients with PTC and patients with benign nodular thyroid disease

	Patients with PTC	Patients with benign nodular thyroid disease
*BRAF* ^V600E^ mutation by ddPCR	92 (76.67%)	0
*BRAF* ^V600E^ mutation by Sanger sequencing	67 (55.83%)	0

### 3.3 Fractional abundance of PTC patients with *BRAF* ^V600E^ mutation

A total of 92 samples with the *BRAF* ^V600E^ mutation were detected by ddPCR at a fractional abundance from 0.28% to 45.40%. Of these, 59 samples (64.13%) were detected at a fractional abundance ≥10%, 8 samples (8.70%) were detected at a fractional abundance from 5% to 10%, and 25 samples (27.17%) were detected at a fractional abundance ≤5% (Table 4).

**Table 4:** *BRAF* ^V600E^ mutations at different fractional abundance by ddPCR

Fractional abundance	Patients
≥10%	59 (64.13%)
5%‐10%	8 (8.70%)
≤5%	25 (27.17%)

Among the 92 positive samples detected by ddPCR, the *BRAF* ^V600E^ mutation was detected in all 59 samples at a fractional abundance ≥10% and in four samples at a fractional abundance from 5% to 10% by Sanger sequencing. Twenty‐five samples were detected at a fractional abundance ≤5% by ddPCR, but Sanger sequencing indicated wild‐type *BRAF* in these samples.

## 4 DISCUSSION

The *BRAF* ^V600E^ mutation is the most common driver mutation in PTC and acts as a useful diagnostic and prognostic marker for PTC.^12^, ^13^, ^14^

Sanger sequencing is the gold standard for detecting the *BRAF* ^V600E^ mutation and is a commonly used method in laboratories. Because of its relatively low sensitivity, the detection of mutations requires a large amount of tumor DNA in the samples. ddPCR is a relatively new method with multiple advantages, such as improved sensitivity and absolute quantification.^15^, ^16^, ^17^, ^18^, ^19^ Anna et al^11^ reported that the ddPCR assay provided accurate fractional abundance estimations at 0.0005% for the *BRAF* ^V600E^ mutation. The advantages and disadvantages of Sanger sequencing and ddPCR are summarized in Table 5.

**Table 5:** Advantages and disadvantages of Sanger sequencing and ddPCR

Platform	Advantages	Disadvantages
Sanger sequencing	Provides sequence information	Low sensitivity Inability to quantify long testing period
ddPCR	High sensitivity Provides absolute quantification Short testing period	Limited to specific mutation Does not provide sequence information

The *BRAF* ^V600E^ mutation was found in 55.83% of PTC patients by Sanger sequencing and 76.67% by ddPCR in the present study. A higher mutation rate was detected by ddPCR. These results were consistent with previous studies that showed a *BRAF* ^V600E^ mutation rate of 29%‐90%.^2^, ^14^, ^20^, ^21^ In the present study, the *BRAF* ^V600E^ mutation was only detected in PTC patients and not in patients with benign nodular thyroid disease, which was also consistent with previous studies.^22^, ^23^

It is important to note that mutations at a fractional abundance ≥10% would have been reported as positive by Sanger sequencing in a clinical setting. Mutations at a fractional abundance from 5% to 10% are difficult to report and would have required confirmation by another method. Mutations at a fractional abundance ≤5% cannot be detected by Sanger sequencing. In this research, good concordance was found between ddPCR and Sanger sequencing in mutations at a high fractional abundance, and all discordant results were found in mutations at a low fractional abundance, which could be explained by the greater sensitivity of ddPCR than Sanger sequencing. Hence, it is possible that Sanger sequencing produced false‐negative results because of the low tumor DNA content and low‐abundance DNA mutations.

In the present study, all specimens were obtained after thyroid surgery, fixed in formalin, and embedded in paraffin. However, high‐quality PTC samples cannot always be acquired in a clinical setting. Most PTC samples for diagnostic testing are obtained by either fine needle aspiration (FNA) or core needle biopsy (CNB) from small tumors with a low tumor content. These specimens may not be suitable for Sanger sequencing. A highly sensitive technique is necessary to detect the *BRAF* ^V600E^ mutation in PTC samples.

In conclusion, ddPCR showed excellent results in the detection of the *BRAF* ^V600E^ mutation, especially in samples with a low tumor DNA content and in low‐abundance DNA mutation samples. ddPCR is a reliable, highly sensitive technique that could replace Sanger sequencing for the detection of the *BRAF* ^V600E^ mutation in PTC samples.
